# The Multifactorial Nature of Early Numeracy and Its Stability

**DOI:** 10.3389/fpsyg.2020.518981

**Published:** 2020-11-04

**Authors:** David Braeuning, Andrew Ribner, Korbinian Moeller, Clancy Blair

**Affiliations:** ^1^LEAD Graduate School & Research Network, University of Tübingen, Tübingen, Germany; ^2^Hector Research Institute of Education Sciences and Psychology, University of Tübingen, Tübingen, Germany; ^3^Leibniz-Institut für Wissensmedien, Tübingen, Germany; ^4^Department of Applied Psychology, New York University, New York, NY, United States; ^5^Department of Psychology, University of Tübingen, Tübingen, Germany; ^6^Centre For Mathematical Cognition, School of Science, Loughborough University, Loughborough, United Kingdom

**Keywords:** early numeracy, basic numerical competences, mathematical abilities, structure, stability, predictor

## Abstract

Early numeracy is a robust predictor of later mathematical abilities. So far, early numeracy has typically been presented as a unitary or two-factorial construct. Nevertheless, there is recent evidence suggesting that it may also be reflected by more basic numerical competences. However, the structure and stability of such a multifactorial model of early numeracy over time has not been investigated yet. In the present study, we used data from a large, longitudinal sample (*N* = 1292) in the United States with assessments of math ability in prekindergarten and kindergarten to evaluate both the factorial structure of early numeracy and its stability over time. Confirmatory factor analysis identified four distinct basic numerical competences making up early numeracy in prekindergarten: patterning/geometry, number sense, arithmetic, and data analysis/statistics. Stability as tested by means of measurement invariance indicated configural invariance of these four factors from prekindergarten to kindergarten. This reflected that early numeracy in kindergarten was made up by the same four basic numerical competences as in prekindergarten and thus seemed rather stable over the course of preschool. These findings may not only have implications for research on numerical cognition but particularly for diagnostic processes or the development of interventions in educational practice.

## Introduction

Basic numerical competences acquired before school-entry are important predictors for later mathematical and educational achievement (e.g., Parsons and Bynner, 2005; Duncan et al., 2007; Jordan et al., 2009, 2010). These competences are often summarized under the broad construct *early numeracy* (e.g., Lembke and Foegen, 2009; Aunio and Niemivirta, 2010). Although it has been suggested that the construct of early numeracy is more accurately represented by multiple distinct basic numerical competences (Dowker, 2008), very few studies have examined the specific structure of basic numerical competences making up early numeracy prior to school entry. Moreover, previous longitudinal studies have typically investigated whether and—if so—which basic numerical competences predict later mathematical achievement operationalized in terms of scores of (standardized) math tests or sometimes math grades (e.g., Parsons and Bynner, 2005; Jordan et al., 2010). As such, they do not describe the development of basic numerical competences themselves, but how they predict other, usually more complex arithmetical and mathematical abilities. In turn, little is known about the stability of basic numerical competences that make up early numeracy and the ways in which they develop over time. Hence, this study aims to evaluate the specific structure of early numeracy by specifying its underlying basic numerical competences and their stability across the transition from preschool (age 5) to kindergarten (age 6).

In the following, we will first give an overview of uni- and multi-dimensional conceptualizations of early numeracy and its dimensionality. We then review previous findings on the stability of numeracy performance and basic numerical competences.

### From a Uni- to a Multi-Dimensional Perspective on Early Numeracy

Previous research on cognitive development has often considered early numeracy as a unidimensional skill. Accordingly, it is subsumed under a single parameter score that reflects performance over a broad range of tasks, covering primarily numerical (e.g., counting, number knowledge, basic calculations; e.g., Jordan et al., 2007, 2009, 2010; Kroesbergen et al., 2009) but also more visual-spatial processes (e.g., recognition of shapes or patterns, geometry; e.g., Jordan et al., 2006; Anders et al., 2012; Polignano and Hojnoski, 2012). However, such a unidimensional conceptualization of early numeracy that averages out contributions of specific basic numerical competences can only provide a rather general measure of early numeracy but not reflect its underlying structure of basic numerical competences adequately. Indeed, Dowker (2008) suggested that children in the preschool years are already capable of performing numerical tasks that require distinct basic competences, suggesting numeracy might be multidimensional even in early childhood prior to formal schooling.

Practically speaking, children develop numerical competences in distinct domains that often correspond to the way that mathematics is taught to them. Content analysis of elementary mathematics textbooks from kindergarten through sixth grade has documented that since the 1960s, mathematics instruction has expanded considerably, in particular in the topics covered (e.g., operations, geometry, patterns, etc.), as well as in the introduction of advanced topics at increasingly earlier grades (Baker et al., 2010). In particular, math education usually differentiates math competences on a conceptual level following content strands (National Council of Teachers of Mathematics, 1989, 2000). These include children’s understanding of (i) properties of numbers, as well as arithmetic operations (e.g., addition, multiplication) and their application to real-world situations, (ii) operating with measurement units like money, time, etc., (iii) geometry from shapes to transformations, (iv) data analysis and statistics as reflected in collecting, organizing, reading, and representing data, and (v) recognition of patterns and functions. These groupings of content areas within mathematics education suggest variation in the types of mathematical competences that children acquire and indicate the potential value of examining variation in distinct domains of mathematical competences when drawing conclusions about achievement in elementary grades.

Previous research following a multidimensional perspective has identified different basic numerical competences to make up the construct of early numeracy. Mostly, two-factorial models have been suggested differentiating, for instance, relational abilities and counting (e.g., Aunio et al., 2004), symbolic and non-symbolic numerical abilities (e.g., Kolkman et al., 2013), or procedural and conceptual abilities (e.g., Ribner et al., 2018). However, these models still reflect rather broad descriptions of early numeracy and few studies have examined further and more specific differentiations that might be more aligned with curricular approaches (e.g., Cirino, 2011). For instance, one such study on the structure of early numeracy comes from an analysis of large-scale assessment data from more than 1,700 5- to 6-year-old children in the Netherlands. In this study, Hirsch et al. (2018) categorized items from an early numeracy test according to the distinct basic numerical competences theoretically underlying the ability to solve each item. The resulting multifactorial models were tested against one-factor and two-factor models; using confirmatory factor analysis, the authors provided evidence for a five-factor structure of early numeracy at the end of kindergarten discerning the factors *patterning, seriation, counting, non-symbolic comparison*, and *symbolic number knowledge*. Additionally, these factors turned out to reliably predict later math performance in a curricular test in grade six. In particular, the authors found a unique association between *non-symbolic comparison, seriation, counting*, and *symbolic number knowledge*, and later mathematical skills, but no unique association for *patterning*.

Hirsch et al. (2018) also discussed that the basic numerical competences underlying early numeracy may depend on the content and range of topics addressed in the respective (large-scale assessment) tests. Consequently, studies considering other data sets based on different tests proposed different models to represent the multifactorial structure of early numeracy. In particular, Purpura and Lonigan (2013) considered data from a preschool assessment that addressed several basic numerical (e.g., counting forward/backward, symbolic and non-symbolic magnitude comparisons, etc.) but not geometric abilities. They found evidence for three highly correlated, yet distinct factors of early numeracy which they termed *numbering* (e.g., counting procedures, subitizing, and estimation), *relational* (e.g., ordinality, number comparison), and (arithmetic) *operational* (e.g., basic addition/subtraction problems) abilities reflecting the structure of early numeracy (see also Purpura and Lonigan, 2015).

This three-factor model was recently replicated and expanded based on data from an assessment of early numeracy that covered a broader range of tasks. In particular, Milburn et al. (2019) observed a four-factor model consisting of the factors *measurement*, *geometry*, *patterning*, and *numeracy*—with the latter conceptualized as a second-order factor that was further differentiated into *numbering*, *relations*, and *operations* as proposed by Purpura and Lonigan (2013). Another four-factor model of early numeracy in kindergarten children was reported by Hellstrand et al. (2020) who differentiated the factors of *symbolic and non-symbolic number knowledge*, *understanding mathematical relations*, *counting*, and *basic arithmetic* (see also Aunio and Räsänen, 2016). Thus, universal characteristics of early numeracy may be most likely derived by integrating results across different tests and different samples.

### Longitudinal Stability of Children’s Numerical Competences

Related to the question of the structure of early numeracy, findings regarding the stability of early numeracy or—more specifically—basic numerical competences underlying early numeracy in children’s development are also limited. In fact—and in part due to the rapid development of numerical competences in early childhood—there is relatively little work in which the same measures were obtained repeatedly in a longitudinal design.

It is clear from the literature that overall (early) numeracy seems highly stable throughout early childhood and beyond (e.g., Bailey et al., 2014; Schmitt et al., 2017). However, in these studies, (early) numeracy was usually assessed by broad (standardized) math tests yielding a single-parameter score that subsumed performance on different numerical tasks. This may be problematic considering the multi-dimensional structure of (early) numeracy (e.g., Hirsch et al., 2018). As such, additional research is needed to better understand the stability of specific basic numerical competences underlying early numeracy and their structure over time. Previous longitudinal research on basic numerical competences has often been limited to very specific competences (e.g., non-symbolic magnitude comparison reflecting the approximate number system, ANS, e.g., Purpura and Simms, 2018) or measures that consist of very few items to describe a broad competence (e.g., counting and cardinality, e.g., van Marle et al., 2014; Purpura et al., 2017). However, the stability of the structure of basic numerical competences underlying early numeracy has been rarely evaluated so far. Even though Hellstrand et al. (2020) as well as Purpura and Lonigan (2013) were able to replicate their models in different age groups (e.g., in younger and older preschool children, Purpura and Lonigan, 2013), longitudinal (i.e., within-person) stability of a multifactorial structure of early numeracy has not yet been evaluated so far and thus remains unclear.

Generally, broader measures of numeracy of pre- and primary school children seem to be rather stable over time, as observed over periods of several months (e.g., Libertus et al., 2013; Chu et al., 2016; Nuutila et al., 2018) or even years (e.g., Aunola et al., 2004; Bailey et al., 2014; Schmitt et al., 2017). For instance, Aunola et al. (2004) obtained numeracy using a curriculum-based test six times over a period of 3 years and found scores to be highly interrelated. Moreover, Bailey et al. (2014) demonstrated that numeracy as assessed by standardized tests was highly stable across both short- (from first to fourth grade of primary school) and long-term periods (from first grade up to the age of 15 years). Using a state-trait model, the authors observed that a high degree of variance in numerical development over time is attributable to trait—rather than state—characteristics. Similarly, Jordan et al. (2007) measured children’s number sense (a composite score of counting, number knowledge, estimation abilities, etc.) at several time points between kindergarten and 1st grade and found it to increase slightly but constantly (see also Jordan et al., 2006). Moreover, the predictive power of number sense measures for later mathematical achievement seemed to be stable throughout primary school (Jordan et al., 2010).

Additionally, previous studies also indicate that the stability of very specific numerical competences is high even in preschool children. For non-symbolic magnitude comparison (as a measure of ANS) some studies reported remarkably high test-retest correlations (e.g., Libertus et al., 2013; Toll et al., 2015; Chu et al., 2016; Purpura and Simms, 2018). For instance, Purpura and Simms (2018) measured ANS twice within 6 months of preschool and observed rather high stability, similar to results for symbolic and non-symbolic magnitude comparison and arithmetic abilities in primary school children (e.g., Göbel et al., 2014).

### The Present Study

In sum, previous research has highlighted that early numeracy is multifactorial in that it is constituted by distinct basic numerical competences. However, studies explicitly investigating the structure of early numeracy are scarce. At the same time, evidence on the longitudinal (i.e., within-person) stability of basic numerical competences making up early numeracy mainly stems from studies which obtained either particularly broad and general or very specific measures of basic numerical competences so far. However, we are not aware of any study evaluating the longitudinal stability of a specific structure of basic numerical competences reflecting early numeracy within the same sample of children over time.

This is of particular interest because in early years of education it is likely that children’s numerical development is dynamic as the numerical concepts become gradually more complex. As such, those basic numerical competences which constitute early numeracy might change. On the other hand, it is also well possible that early numeracy is a rather stable construct as its components reflect very basic building blocks of children’s numerical competences reflected in curricular content strands (i.e., number sense and operations; measurement; geometry; data analysis and statistics; and patterning). Therefore, the aims of the present study were to investigate (i) the structure of early numeracy and (ii) to evaluate the stability of this structure over time in preschool children.

To pursue these aims, we relied on data from the Family Life Project (FLP), a large population-based prospective longitudinal study of children and families in predominantly low-income, non-urban communities in the United States. In addition to numerous aspects of child and family functioning, the FLP dataset contains data from the standardized math assessment developed for the Early Childhood Longitudinal Study-Kindergarten Cohort of 1998 (ECLS-K). Given that prior research and practice in math education indicated the need for a detailed *a priori* differentiation of numerical-mathematical competences (e.g., Aunio et al., 2006), we considered a confirmatory approach as particularly valuable to analyze the structure of early numeracy. Moreover, we tested whether this structure is stable across time by evaluating its validity to account for children’s performance on the same test 1 year later.

## Materials and Methods

### Participants

The original sample that made up the Family Life Project from which data are drawn was recruited when children were 2 months of age and comprised *N* = 1,292 children recruited to be representative of two of the four major geographical areas of high child rural poverty in the United States. Complex sampling procedures were used to recruit representative samples of non-urban areas of Pennsylvania and North Carolina, with intentional over sampling of low-income families and families of African American ethnicity. Five years later at PreK, over 70% of children (*n* = 911) participated in assessment, and in kindergarten over 80% of children (*n* = 1056) participated. We anticipate some of the difference between the two testing sessions was that not all children were enrolled in center-based care for PreK and were therefore more difficult to access whereas in kindergarten almost all children were enrolled in a school. Seventy percent of families had an average income of less than 200% of the poverty line. Additionally, 40% of mothers had a high school education (12 years of schooling) or less, while only 16% had at least 4 years of postsecondary education. A little more than half of the sample is White (57%) with the remainder of African American descent. Further details are available elsewhere (Vernon-Feagans et al., 2013).

### Procedures

When children were approximately 60 months of age (*M* = 60.16 months, *SD* = 3.29) children were visited at their preschools by a trained data collector (or at home if they were not enrolled in center- or school-based care) to obtain the measure of early numeracy. In the spring of the child’s kindergarten year (*M*_*age*_ = 71.40 months, *SD* = 3.36), they were again visited at their school by a trained data collector to re-assess early numeracy using the same test.

### Measure of Math Ability

The ECLS-K math assessment was used to test children’s early numeracy. The ECLS-K assessment uses a routing system to minimize administration time and most accurately assess their ability, and is a reliable and valid measure whose psychometric properties have been described elsewhere (Rock and Pollack, 2002). The same assessment was used in both pre-kindergarten (PreK) and kindergarten. All participants receive a series of 14 routing items. If participants scored 8 or lower on routing items, they are directed to the “low” block; if higher than 8, an additional 4 routing items were administered. If participants correctly respond to between 9 and 11 items, they were routed to the “medium” block; if 12 or higher, they were routed to the “high” block. The low block had 18 items plus the 14 routing items, the medium block had 25 items plus the 18 routing items, and the high block had 32 items plus the 18 routing items. Children were routed to high, medium, or low blocks on the basis of the number of items they got correct on the routing section of the assessment (all other things being equal). In particular, there was no adjustment for any child characteristics (e.g., age and sex) such that any child had equal probability of being routed to any block. In PreK, 93.2% of participants (*N* = 849) were routed to the “low” block, 5.5% of participants (*N* = 50) were routed to the “medium” block, and 1.3% of participants (*N* = 12) were routed to the “high” block. In kindergarten, 51.8% of participants (*N* = 537) were routed to the “low” block in PreK, 30.2% of participants (*N* = 319) were routed to the “medium” block, and 18.9% of participants (*N* = 200) were routed to the “high” block.

### Analytic Strategy

We conducted confirmatory factor analysis (CFA) to analyze the multifactorial structure of basic numerical competences underlying early numeracy. In particular, we specified and evaluated a one-factor model representing early numeracy as a unitary construct and compared it to a multifactorial (six-factor) model in which items from the ECLS-K were classified based on the basic numerical competences necessary to solve each item. The categories for item coding (six basic numerical competences) were derived from the psychometric report of the ECLS-K (Rock and Pollack, 2002). This classification is mainly based on curriculum standards and reflects the way in which the ECLS-K was designed. Therefore, it takes the specific characteristics of this test into account while it also shows structural and conceptual similarities to previous multifactorial models of early numeracy. Moreover, it covers distinct basic numerical competences that have already been investigated in previous early numeracy research (for an overview see Table 1 in Hirsch et al., 2018). To evaluate how well the data fit the theorized models, we considered the cutoff criteria presented by Hu and Bentler (1999): A well-fitting model was expected to have a Comparative Fit Index (CFI) > 0.95, and Root Mean Squared Error of Approximation (RMSEA) < 0.08. Models were estimated in Mplus (Muthén and Muthén, 2017) and used the Weighted Least Squared Means and Variances (WLSMV) estimator. Prior research suggested WLSMV is appropriate for ordinal variables, and is less biased than are other estimators (Li, 2016). In a second step, measurement invariance was tested to establish whether the same constructs could be established 1 year later in kindergarten to evaluate the stability of early numeracy. Adequate model fit was determined by use of a chi-square difference test and whether CFI changed more than 0.002 (Meade et al., 2008).

Participants were included in analyses if they took part in the PreK wave of data collection and were routed to the “low” block (*N* = 849; 93.2%). Missing data at the re-test in Kindergarten was accounted for using Full Information Maximum Likelihood estimation. This approach takes into account the covariance matrix for all available data on the independent variables to estimate parameters and standard errors and provides more accurate estimates of regression coefficients than do listwise deletion or mean replacement (Enders, 2011).

## Results

### Descriptive Statistics

Descriptive statistics for items from the ECLS-K math assessment are shown in Table 1. Correlations between all variables are shown in Table 2.

**TABLE 1:** Descriptive analyses for items from ECLS-K math assessment.

		Pre-K				Kindergarten			
			
Item	Item type	*N*	% correct	*SD*	Factor loading	*N*	% correct	*SD*	Factor loading
17	Arithmetic	846	0.31	0.46	0.57	830	0.51	0.50	0.85
26	Arithmetic	846	0.42	0.49	0.51	440	0.65	0.48	0.36
11	Arithmetic	846	0.16	0.37	0.57	440	0.49	0.50	0.34
25	Arithmetic	846	0.18	0.39	0.47	440	0.20	0.40	0.29
31	Data Analysis/statistics	846	0.57	0.50	0.92	440	0.83	0.37	0.90
30	Data Analysis/statistics	846	0.48	0.50	0.85	440	0.75	0.43	0.93
18	Number sense	846	0.76	0.43	0.89	440	0.96	0.20	0.63
19	Number sense	846	0.63	0.48	0.90	440	0.91	0.29	0.56
5	Number sense	846	0.43	0.50	0.80	830	0.87	0.33	0.76
20	Number sense	846	0.20	0.40	0.80	440	0.51	0.50	0.45
4	Number sense	846	0.28	0.45	0.68	830	0.76	0.43	0.54
6	Number sense	846	0.11	0.32	0.60	830	0.61	0.49	0.78
16	Number sense	846	0.86	0.35	0.63	440	0.96	0.19	0.36
7	Number sense	846	0.11	0.31	0.56	830	0.61	0.49	0.85
15	Number sense	846	0.94	0.23	0.61	440	0.98	0.14	0.27
9	Number sense	846	0.14	0.35	0.47	830	0.62	0.49	0.75
32	Number sense	846	0.37	0.48	0.25	440	0.40	0.49	0.17
29	Patterning	846	0.49	0.50	0.57	440	0.69	0.46	0.32
2	Patterning	846	0.73	0.45	0.51	830	0.91	0.29	0.59
8	Patterning	846	0.58	0.49	0.57	830	0.82	0.39	0.64
1	Patterning	846	0.53	0.50	0.47	830	0.76	0.43	0.61
22	Patterning	846	0.44	0.50	0.41	440	0.61	0.49	0.36
3	Patterning	846	0.41	0.49	0.30	830	0.65	0.48	0.56
24	Patterning	846	0.38	0.48	0.30	440	0.57	0.50	0.24
10	Excluded	846	0.01	0.12	N/A	830	0.17	0.38	N/A
12	Excluded	846	0.15	0.36	N/A	830	0.31	0.46	N/A
13	Excluded	846	0.05	0.23	N/A	830	0.22	0.41	N/A
14	Excluded	846	0.02	0.14	N/A	830	0.14	0.34	N/A
21	Excluded	846	0.90	0.30	N/A	440	0.96	0.20	N/A
23	Excluded	846	0.33	0.47	N/A	440	0.52	0.50	N/A
27	Excluded	846	0.04	0.20	N/A	440	0.07	0.25	N/A
28	Excluded	846	0.11	0.32	N/A	440	0.18	0.39	N/A

**TABLE 2:** Correlations among latent variables.

		1	2	3	4	5	6	7	8
1	Patterning PreK	–							
2	Number Sense PreK	0.70***	–						
3	Arithmetic PreK	0.89***	0.66***	–					
4	Data Analysis/Statistics PreK	0.65***	0.57***	0.71***	–				
5	Patterning K	0.86***	0.57***	0.70***	0.58***	–			
6	Number Sense K	0.69***	0.77***	0.63***	0.53***	0.71***	–		
7	Arithmetic K	0.71***	0.54***	0.84***	0.57***	0.82***	0.74***	–	
8	Data Analysis/Statistics K	0.13	0.18**	0.32**	0.25***	0.42***	0.39***	0.48***	–

***p < 0.001, **p < 0.01.

### Confirmatory Factor Analysis of Pre-kindergarten Basic Numerical Competences

In line with theoretical considerations and the psychometric report from the ECLS-K assessment items were coded as assessing one of six basic numerical competences: Patterning (6 items), Number Sense (12 items), Arithmetic (7 items), Geometry (3 items), Measurement (2 items), and Data Analysis/Statistics (2 items). Table 3 provides a more detailed description of the items. In our first model, we were interested to test whether there was sufficient distinction of separate constructs to justify the operationalization of six separate numerical competencies. To do so, we first tested whether all items loaded onto a single factor. The resulting model did not fit the data well, χ^2^ = 1021.04, *p* < 0.001; RMSEA = 0.038, 90% CI [0.035, 0.041]; CFI = 0.904. We then tested a second model in which the data were fit to the six purported constructs. The resulting model also did not fit the data at our desired levels, χ^2^ = 761.62, *p* < 0.001; RMSEA = 0.029, 90% CI [0.025, 0.032]; CFI = 0.946; however, the model fit was improved significantly over the single-factor model, χ^2^ = 261.61, *p* < 0.001. The model was further specified: Items that did not load onto any of the constructs at a level of *p* = 0.10 or lower, as well as items without sufficient variance in responses (i.e., items which were correctly/incorrectly solved by almost all children) were dropped (8 items in total). Additionally, modification indices suggested items in the *measurement* (1 item) and *geometry* (2 items) categories loaded onto the *patterning* category, and one item from the *number sense* category loaded onto the *arithmetic* category. These modifications seemed theoretically justified. In particular, structural similarities were found in the geometry and patterning items as both required visuospatial recognition of shapes and patterns and the measurement item was presented in a similar way as patterning and geometry items (i.e., children had to select the correct solution from a set of four alternatives). The number sense item was conceptualized as assessing counting, but it required addition of three sets of objects which indeed seemed related to arithmetic. We therefore decided to re-specify the model accordingly. The resulting 4-factor model fit the data well, χ^2^ = 474.26, *p* < 0.001; RMSEA = 0.033, 90% CI [0.029, 0.038], CFI = 0.962. Because the updated model had items missing, model fit could not be formally compared to the 6-factor model using a log-likelihood difference test because models were no longer nested; however, model fit for the 4-factor model was adequate according to conventional norms whereas it was not for the 6-factor model. Fit indices for all models are provided in Table 4.

**TABLE 3:** Item descriptions.

Item	Item type	Item description
11	Arithmetic	(Object-based) addition^a^
17	Arithmetic	(Object-based) addition
25	Arithmetic	(Object-based) addition
26	Arithmetic	(Object-based) subtraction
30	Data Analysis/statistics	Graph reading^a^
31	Data Analysis/statistics	Graph reading
4	Number sense	Counting forward
5	Number sense	Identify a number (symbolic number knowledge)
6	Number sense	Identify a number (symbolic number knowledge)
7	Number sense	Identify the *n*th object (ordinality)
9	Number sense	Complete a number series (seriation/number order)
15	Number sense	Count a set
16	Number sense	Numeral recognition^a^
18	Number sense	Identify a written number (symbolic number knowledge)
19	Number sense	Identify a written number (symbolic number knowledge)
20	Number sense	Identify a written number (symbolic number knowledge)
32	Number sense	Estimation (non-symbolic)
1	Patterning	Match a pattern of objects/shapes to a different pattern from a set^a^
2	Patterning	Match a pattern of objects/shapes to a different pattern from a set
3	Patterning	Match a pattern of objects/shapes to a different pattern from a set
8	Patterning	Choose a shorter/larger object from a set (geometry/length concept)
22	Patterning	Match a pattern of objects/shapes to a different pattern from a set
24	Patterning	Match a geometric shape to a different shape from a set (geometry)
29	Patterning	Measure length of an object (measurement)
12	Excluded (arithmetic)	(Object-based) subtraction
13	Excluded (arithmetic)	(Object-based) addition
27	Excluded (arithmetic)	(Object-based) addition
28	Excluded (arithmetic)	(Object-based) subtraction
21	Excluded (geometry)	Recognize a geometric shape
14	Excluded (measurement)	Operate with money (measurement)
10	Excluded (number sense)	Complete a number series (seriation/number order)
23	Excluded (patterning)	Match a pattern of objects/shapes to a different pattern from a set

**FIGURE 1:**
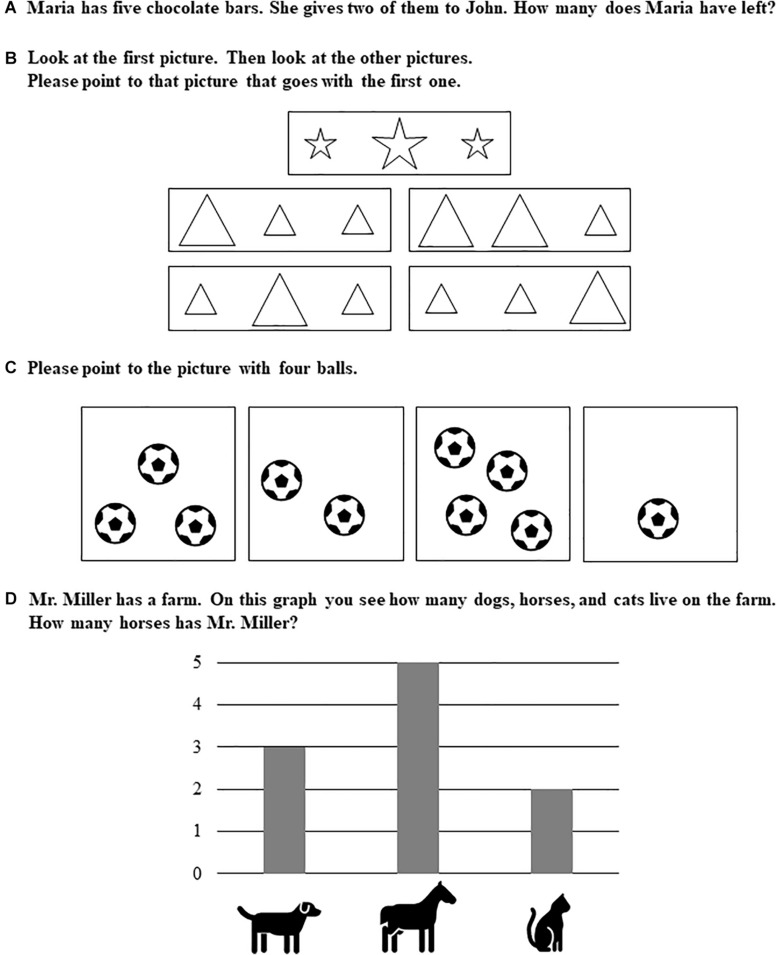
Generic example items for **(A)** arithmetic, **(B)** patterning/geometry, **(C)** number sense, and **(D)** data analysis/statistics. Instructions were read out by the investigators while the items were shown to the children on a separate sheet in an open-bound spiral notebook.

*^*a*^see Figure 1 for a generic example.*

**TABLE 4:** Model fit information.

Model	χ^2^	df	CFI	RMSEA [90%CI]
One factor	1021.04	464	0.904	0.038 [0.035, 0.041]
Six factor	761.62	449	0.946	0.029 [0.025, 0.032]
Four factor	474.3	246	0.962	0.033 [0.029, 0.038]

The 4-factor model included factors for *patterning and geometry*, *number sense*, *arithmetic*, as well as *data analysis and statistics* (see Figure 1 for illustrating example items, see Table 1 for item properties). Factor loadings for each factor are presented in Table 1. The variances of latent variables were significant (patterning, σ^2^ = 0.23, *p* < 0.001; number sense, σ^2^ = 0.43, *p* < 0.001; arithmetic, σ^2^ = 0.26, *p* < 0.001; data analysis/statistics, σ^2^ = 0.71, *p* < 0.001). The four latent variables were correlated with one another, and correlations among latent variables are presented in Table 2. Children showed the highest levels of understanding *patterning and geometry* (*M* = 53.82% correct) and *data analysis/statistics* (*M* = 52.60% correct), then *number sense* (*M* = 46.76% correct), and *finally arithmetic* (*M* = 29.19% correct).

### Stability of Basic Numerical Competences Underlying Early Numeracy

To test the validity and stability of the numerical competences established in PreK, a series of models were run to test longitudinal measurement invariance of numerical competences in kindergarten. We first tested configural invariance to examine whether the items that represented the identified constructs in PreK continued to do so in kindergarten. A confirmatory model in which the same four factors (i.e., patterning/geometry, number sense, arithmetic, and data analysis/statistics) were simultaneously estimated in PreK and kindergarten. The model fit the data well, χ^2^ = 1531.184, *p* < 0.0001; RMSEA = 0.023, 90% CI [0.021, 0.026], CFI = 0.950, such that configural invariance could be concluded indicating that the same items represented the identified constructs in PreK and kindergarten.

Metric invariance was then tested to examine the relative contribution of the items within factors, in that the coefficients of items in each factor were set to be equal across administration (that is, in PreK and kindergarten). Model fit was acceptable, χ^2^ = 1935.951, *p* < 0.0001; RMSEA = 0.031, 90% CI [0.029, 0.033], CFI = 0.910; however, the chi-square test of model difference was significant (χ^2^ = 204.604, *p* < 0.001) and CFI changed markedly more than 0.002, indicating metric invariance was not held.

## Discussion

Prior empirical work has suggested that early numeracy might be better represented as a multidimensional construct made up of distinct basic numerical competences than a single unitary construct (e.g., Dowker, 2008). However, multidimensional conceptualizations are rare. Additionally, evidence on the longitudinal (i.e., within-person) stability of a specific structure of basic numerical abilities underlying early numeracy over time is limited. The present study aimed at complementing prior research by evaluating the longitudinal stability of the structure of basic numerical competences in a large longitudinal data set of young children in the United States. Primary aims were (i) to test the structure of basic numerical competences constituting early numeracy in 5-year-old children and (ii) to evaluate the stability of this structure over 1 year from PreK through kindergarten. In the following, we will discuss these aspects in turn.

### Structure of Basic Numerical Competences Underlying Early Numeracy

Early numeracy has been typically considered a unitary or two-factorial construct in previous studies (e.g., Aunio et al., 2004; Jordan et al., 2010). However, there is evidence also using large-scale assessment data that early numeracy in preschool years may be constituted by more than two basic numerical competences (Purpura and Lonigan, 2013; Hirsch et al., 2018; Milburn et al., 2019; Hellstrand et al., 2020). In these studies, items from large-scale assessments of early numeracy were used to specify and evaluate multifactorial models by means of confirmatory factor analysis. However, these models differed in content and structure. In particular, Purpura and Lonigan (2013) established a three-factor model of early numeracy with factors for *numbering*, *relation*, and *operation* competences. Milburn et al. (2019) extended this model by adding another three distinct factors for *measurement*, *geometry*, and *patterning* competences. Another four-factor model with *symbolic/non-symbolic number knowledge*, *numerical relations*, *basic arithmetic*, and *counting* competences was recently presented by Hellstrand et al. (2020), and Hirsch et al. (2018) substantiated a five-factor model reflecting *patterning*, *seriation*, *non-symbolic comparison*, *counting*, and *symbolic number knowledge* abilities. In contrast, the present study using the ECLS-K math assessment identified early numeracy as constituted by four basic numerical competences reflected in a confirmatory factor analytic approach. The one- and six-factor confirmatory models evaluated did not yield adequate model fit; rather, our results provided evidence for four basic numerical competences underlying early numeracy as assessed by the ECLS-K math assessment: (i) *patterning and geometry*, (ii) *number sense*, (iii) *arithmetic*, as well as (iv) *data analysis and statistics*.

Comparing our results with those of previous studies revealed that the basic numerical competences specified in the different models were quite similar indeed. Most obviously, we also found a factor for basic *arithmetic operations* similar to Purpura and Lonigan (2013), Milburn et al. (2019), and Hellstrand et al. (2020), and a factor for *patterning* (which here also included *geometry* and *measurement*) as did Hirsch et al. (2018) and Milburn et al. (2019). Although several factors of models reported in other studies did not directly correspond to those observed in the present study, a more detailed comparison of item contents from this and previous studies revealed (Table 3) that they seem to be in part subsumed in our *number sense* factor. In particular, *number sense* was mainly assessed by symbolic number knowledge and counting items and therefore largely overlaps with the *numbering* factor in Purpura and Lonigan (2013) and Milburn et al. (2019). Additionally, it comprised a few items on seriation, ordinality, and estimation which overlaps with content of the *relations* factor in the models of Purpura and Lonigan (2013) and Milburn et al. (2019), or the *relations* and *symbolic/non-symbolic number knowledge* factors in the model of Hellstrand et al. (2020). Furthermore, our *number sense* factor may reflect a conjunction of four factors of the model by Hirsch et al. (2018), namely *counting*, *seriation*, *symbolic number knowledge*, and *non-symbolic comparison*.

Despite these significant commonalities, our model of early numeracy differed in at least three notable aspects from previously identified multifactorial models. First, as already indicated above, *number sense* described a rather broad factor compared to more specific numerical competences the other studies specified. However, this may be due to the fact that the ECLS-K math assessment was explicitly designed to measure number sense broadly, which made it difficult to identify more specific competences based on the number sense items as too few items were available. For instance, we might have further specified a specific factor for seriation, but only one item actually addressed seriation in the ECLS-K assessment. As such, it was more appropriate to summarize such (single) items under a more general *number sense* factor.

Second, a similar reason may also explain why we did not find distinct factors for *patterning*, *geometry*, and *measurement* as did Milburn et al. (2019). In particular, their model comprised four patterning, seven geometry, and six measurement items, while we identified only five patterning, two geometry and one measurement items. Accordingly, limited variance on *geometry* and *measurement* in our data may have been best explained by *patterning*. Additionally, geometry and measurement items were structurally very similar to the patterning items.

Lastly, our model suggests that *data analysis and statistics* seemed to represent another distinct basic numerical competence that may already emerge in preschool years. This competence describes children’s ability to read and draw inferences of graphical representations of data. To the best of our knowledge, *data analysis and statistics* has not yet been reported in other multifactorial models of preschoolers’ early numeracy.

At the same time, however, all differences between models discussed here may not be unexpected as different assessments of early numeracy with (partially) different mathematical-numerical content assessed may lead to the identification of different basic numerical competences that constitute the construct of early numeracy (cf. Hirsch et al., 2018). This may be particularly so given that the large-scale assessment tests considered in some of the studies, including this one, originally intended to reflect a broad assessment of early numeracy as it was defined in curricular standards of different educational systems (e.g., the Netherlands vs. United States). However, they were not designed to explicitly measure a universal structure of specific basic numerical competences. Nevertheless, we think that it is these comparisons across studies on different samples and different assessments that offer a promising way to gain a comprehensive multidimensional view on early numeracy.

### Stability of Basic Numerical Competences Constituting Early Numeracy

After substantiating the four-factor structure of early numeracy assessed in the ECLS-K math assessment, we evaluated the stability of this structure by testing measurement invariance of the factor structure with a subsample of children that were assessed twice on the same test from PreK to kindergarten. Our analyses revealed configural but not metric invariance, indicating that we were able to identify the same four factors (with the same items) of early numeracy in kindergarten as in PreK, but within that year the relative contributions of items to the factors (i.e., factor loadings) changed. In other words, when children became older some items became stronger (or weaker) indicators of the respective basic numerical competences. Most likely, this reflects that children became more proficient in math and were better able to solve the respective numerical tasks in kindergarten than in PreK. This is also reflected in the smaller number of children routed to the low block in the ECLS-K math assessment. Importantly, however, the four basic numerical competences constituting early numeracy in PreK remained stable to kindergarten with all factor loadings of indicators on a significant and meaningful level. Taken together, these findings suggest that a structure of early numeracy that consists of four correlated factors (i.e., *patterning and geometry, number sense, arithmetic*, as well as *data analysis and statistics*) continues to be refined and improved over time.

However, we cannot conclude whether the factor structure we established here remains stable beyond the preschool years. In particular, the curricula to which children were exposed in PreK and kindergarten were likely more comparable than those of kindergarten and first or second grade; indeed, prior investigations have suggested that many kindergarten teachers spend the majority of their time teaching students what they already learned in preschool (Engel et al., 2013). As such, it is possible that after the beginning of formal schooling when numerical/mathematical content becomes increasingly complex and math instruction more formal, children alter and restructure their early numeracy more substantially. The fact that we did not observe metric invariance of the evidenced four-factor structure may already indicate substantial changes to take place. However, some prior evidence suggests stability of early numeracy at least through the early years of education (e.g., Hellstrand et al., 2020, established their four-factor model in samples of kindergarten, first, and second grade children). Nevertheless, it should be subject to future research to investigate a multifactorial structure of basic numerical competences and follow its development longitudinally across a longer period of time than it was done in the present study. As such, the four basic numerical competences we established in the present study seem to be an essential foundation of children’s early numeracy over the course of preschool. This may have practical and theoretical implications.

Research on intelligence has shown that analyzing the contributions of specific cognitive abilities to school achievement is more informative than considering only a general *g*-factor of intelligence (e.g., Gustafsson and Balke, 1993; Calvin et al., 2010). Similar to these results, the present study highlights the need and value of a more differentiated view on early numeracy in children (cf. Cowan and Powell, 2014). Information on specific basic numerical competences that make up children’s early numeracy as well as their stability during preschool is not only essential for research on numerical cognition, but also in broader educational contexts. For instance, when it comes to diagnose children with mathematical learning difficulties it is important to identify their problems and deficits as early and as specific as possible to initiate targeted interventions (e.g., Geary et al., 2009). This study provides empirical evidence that may help to improve both the diagnostic process itself but also the development of subsequent interventions as it allows for the specification of basic numerical competences making up early numeracy. Based on this, it should be possible to develop diagnostic tools to specifically assess and intervene upon these basic numerical competences. As the present study is among the first of its kind, it must be acknowledged that implications for education are tentative, and further research is required to substantiate both the generalizability and longitudinal relevance of the present findings.

### Limitations and Perspectives

The present study was inspired by the multifactorial model of early numeracy proposed by Hirsch et al. (2018). So far, previous research identified several basic numerical competences often using different tests and sometimes different labels but more or less corresponding to each other when considering underlying basic numerical competences (for an overview see Table 1 in Hirsch et al., 2018). As such, it would be desirable to develop a consensual conceptualization of early numeracy which serves as a framework in future research on basic numerical competences and their long-term relevance.

In the present study, early numeracy was found to be constituted by the four basic numerical competences *patterning and geometry, number sense, arithmetic*, as well as *data analysis and statistics*. As such, we propose that patterning and geometry are also important domains of early numeracy. It should be noted, however, that this goes beyond previous studies that primarily focused on number-related and operational content (e.g., counting, cardinality understanding, addition/subtraction, etc.) when conceptualizing early numeracy (e.g., Purpura et al., 2011; Purpura and Lonigan, 2013; Nelson and McMaster, 2018). Nevertheless, consideration of patterning and/or geometry as important to early numeracy is in line with other studies (e.g., Jordan et al., 2006; Clements and Sarama, 2011; Polignano and Hojnoski, 2012; Rittle-Johnson et al., 2015; Hirsch et al., 2018; Milburn et al., 2019). Moreover, it is also in line with curricular strands on early math education which typically incorporate patterning and early geometry (National Council of Teachers of Mathematics, 1989, 2000).

Moreover, there are further limitations to be considered when interpreting the current results, which may – at the same time – provide interesting avenues for future research. First, we did neither evaluate within-sample effects (e.g., child gender) as we expect the stability of constructs should not vary as a result of demographic characteristics, nor did we consider influences of other domain-general abilities (e.g., language ability and executive functions) on the development of basic numerical competences in our model. Prior research suggested significant interrelations of these variables and several (general or specific) measures of math ability (e.g., Blair et al., 2008; Praet et al., 2013). However, one might expect that they influence specific basic numerical competences differentially. For instance, Purpura et al. (2017) investigated how different components of executive functions (i.e., response inhibition/inhibitory control, cognitive flexibility, and working memory) predicted performance on various tasks on early numeracy. Response inhibition and cognitive flexibility turned out to predict, among others, measures that would correspond to the *number sense* factor in our model (e.g., subitizing, counting, number ordering, and cardinality). In contrast, working memory primarily predicted performance in tasks that required to execute multiple steps or keeping track of intermediate results (e.g., computations). As such, it may mostly be related to the *arithmetic* operations factor of the present model. Similarly, language ability may be most strongly related to competences, which were assessed using word problems or other largely text-based items, that is, *arithmetic* and *data analysis/statistics* in the current model. Nevertheless, we can only speculate about these potential influences so far and it should be investigated in future studies to which degree variance in specific basic numerical competences may be actually explained by domain-general variables. At the same time, however, it should also be noted that using large-scale assessment data for the purpose of secondary data analysis may be often constrained insofar as further potentially interesting variables (e.g., covariates, further indices of achievement) were not addressed during data collection.

Second, the results of the present study might not be generalizable to a wider population. While we leverage quite a large sample, findings may primarily apply to a certain population due to the sample characteristics (i.e., children from low-income families living mainly in non-urban regions in the United States). In particular, we were able to investigate the stability of the factor structure of early numeracy only in those children who were routed to the low-ability block of tasks in the math assessments in PreK and kindergarten as this was the largest group in the longitudinal sample. As such, early numeracy might be less stable in children with higher abilities or steeper learning curves in math. It is conceivable, for instance, that those children develop further numerical competences during preschool due to a differentiation of their number sense abilities (e.g., into symbolic and non-symbolic numerical abilities). This process might be delayed in the low-performing children we considered in the present study.

Finally, as noted earlier, future research may continue to investigate the multifactorial structure of early numeracy longitudinally across longer time periods. This would further allow to evaluate the longitudinal relevance of specific early numerical and later mathematical competences. In particular, prior research primarily specified the predictive power of early basic numerical competences for later general math achievement. For instance, early symbolic numerical competences (e.g., symbolic number knowledge) were shown to predict later math achievement (see e.g., Schneider et al., 2017 for a review). Additionally, Hirsch et al. (2018) found all but one of the competences specified in their 5-factor model of early numeracy to predict math achievement in grade 6. However, it is currently not clear how basic numerical competences establishing a multifactorial structure of early numeracy may relate to a differentiated multifactorial structure of basic numerical competences and/or more advanced mathematical competences (e.g., fraction understanding) established later within the same individuals (i.e., beyond the period of 1 year we covered in this study). Investigating this in more depth would provide more detailed knowledge on the long-term development of basic numerical competences and of potential variation in their interrelations as well as their relation to more advanced mathematical abilities.

Moreover, most recent studies in basic numerical cognition research face the issue of rather small sample sizes. In particular, Kolkman et al. (2013) argued the need for replications of findings using large-scale data. In this study, we specified the structure and stability of latent basic numerical competences underlying a broad curriculum-based assessment and discussed remarkable overlap with prior studies taking a similar approach. We thus see a specific advantage of confirmatory analyses considering large-scale assessment data of early numeracy in general and when it comes to evaluate basic numerical competences with different tests in different samples in particular.

## Conclusion

To the best of our knowledge, this study is the first attempt to replicate a multifactorial model of early numeracy using large-scale assessment data across the PreK and kindergarten years. Importantly, we found further evidence that early numeracy in preschool children is constituted by different basic numerical competences. In particular, we found early numeracy to be reflected by the following four basic numerical competences: (i) *patterning and geometry*, (ii) *number sense*, (iii) *arithmetic*, as well as (iv) *data analysis and statistics*. Although labeled differently, we were able to replicate most factors proposed in prior studies on the multifactorial structure of early numeracy. Moreover, we provided first evidence for the stability of the structure of basic numerical competences constituting early numeracy from PreK to kindergarten. This highlights the role of early numeracy and its underlying basic numerical competences as an important foundation for later numerical-mathematical development. The present findings imply, that preschool education should recognize the multidimensional nature of early numeracy and specifically foster children’s mastery of basic numerical competences.

## Family Life Project Key Investigators

The Family Life Project Key Investigators include Lynne Vernon-Feagans, The University of North Carolina, Mark Greenberg, The Pennsylvania State University, Martha Cox, The University of North Carolina, Clancy Blair, New York University, Peg Burchinal, The University of North Carolina, Michael Willoughby, The University of North Carolina, Patricia Garrett-Peters, The University of North Carolina, Roger Mills-Koonce, The University of North Carolina.

## Data Availability Statement

The datasets generated for this study will not be made publicly available as some restrictions will apply. Data are from the Family Life Project study whose authors may be contacted at https://flp.fpg.unc.edu/.

## Ethics Statement

The studies involving human participants were reviewed and approved by Institutional Review Boards of The University of North Carolina and The Pennsylvania State University. Written informed consent to participate in this study was provided by the participants’ legal guardian/next of kin.

## Author Contributions

DB, AR, and KM conceptualized the study. DB and AR wrote the main manuscript text. AR performed the statistical analyses. CB and KM provided critical editorial feedback and thoughtful revision to the text. All authors reviewed and approved the manuscript as written.

## Conflict of Interest

The authors declare that the research was conducted in the absence of any commercial or financial relationships that could be construed as a potential conflict of interest.
